# COVID-19: A Review on the Novel Coronavirus Disease Evolution, Transmission, Detection, Control and Prevention

**DOI:** 10.3390/v13020202

**Published:** 2021-01-29

**Authors:** Anshika Sharma, Isra Ahmad Farouk, Sunil Kumar Lal

**Affiliations:** 1 School of Science, Monash University Malaysia, Bandar Sunway 47500, Selangor DE, Malaysia; Anshika.Sharma@monash.edu (A.S.); isra.farouk@monash.edu (I.A.F.); 2 Tropical Medicine & Biology Multidisciplinary Platform, Monash University Malaysia, Bandar Sunway 47500, Selangor DE, Malaysia

**Keywords:** COVID-19, 2019-nCoV, emerging infectious disease, zoonotic cycle, positive-sense RNA viruses, evolution

## Abstract

Three major outbreaks of the coronavirus, a zoonotic virus known to cause respiratory disease, have been reported since 2002, including SARS-CoV, MERS-CoV and the most recent 2019-nCoV, or more recently known as SARS-CoV-2. Bats are known to be the primary animal reservoir for coronaviruses. However, in the past few decades, the virus has been able to mutate and adapt to infect humans, resulting in an animal-to-human species barrier jump. The emergence of a novel coronavirus poses a serious global public health threat and possibly carries the potential of causing a major pandemic outbreak in the naïve human population. The recent outbreak of COVID-19, the disease caused by SARS-CoV-2, in Wuhan, Hubei Province, China has infected over 36.5 million individuals and claimed over one million lives worldwide, as of 8 October 2020. The novel virus is rapidly spreading across China and has been transmitted to 213 other countries/territories across the globe. Researchers have reported that the virus is constantly evolving and spreading through asymptomatic carriers, further suggesting a high global health threat. To this end, current up-to-date information on the coronavirus evolution and SARS-CoV-2 modes of transmission, detection techniques and current control and prevention strategies are summarized in this review.

## 1. Introduction

Coronaviruses, belonging to the Coronaviridae family, cause respiratory infection in mammals, such as bats, camels and masked palm civets, and in avian species [1,2]. Symptoms and tissue tropism of coronavirus infection can vary across different host species [3]. In humans, coronavirus infections may be asymptomatic or accompanied by fever, cough, shortness of breath and gastrointestinal irritation [4,5]. In certain cases, particularly in elderly and immunocompromised individuals, coronavirus infections may lead to severe pneumonia and subsequently, the death of the patient [6].

To date, there have been three major coronavirus outbreaks reported, with the most recent epidemic being the spread of the 2019 novel coronavirus (2019-nCoV, or more recently named SARS-CoV-2), which is known to cause the Coronavirus Disease-2019 (COVID-19) [7,8]. On 30 January 2020, the World Health Organization (WHO) declared a global emergency over the novel coronavirus outbreak in Wuhan, a city located in China’s Hubei province [9]. On 24 February 2020, the WHO acknowledged that the SARS-CoV-2 has the potential to spread globally and cause a pandemic outbreak [10,11]. Subsequently, on 11 March 2020, the WHO declared the COVID-19 a pandemic [12]. In this review, we aim to provide consolidated up-do-date available information on this rapidly growing pandemic, including the evolution, transmission, detection and control and prevention strategies against SARS-CoV-2.

## 2. Evolution of the Coronavirus

Coronaviruses were named after the Latin word corona, meaning crown or halo, owing to their crown-like spikes on the surface as seen when viewed under an electron microscope [13]. Coronaviruses are enveloped viruses containing a non-segmented, single-stranded, positive-sense RNA genome of approximately 32 kilobases, thus making it the largest known genome for an RNA virus [14,15,16,17,18,19]. Coronaviruses belong in the subfamily coronavirinae of the coronaviridae family, in the order of nidovirales. The Coronavirinae subfamily consists of four genera: alphacoronavirus, betacoronavirus, deltacoronavirus and the gammacoronavirus, with the SARS-CoV-2 strain being classified under the betacoronavirus genus based on the genome sequence analysis [16,17]. The coronavirus genome is known to have a 5′ cap and a 3′ poly (A) tail; therefore, upon infecting the host cell, the genome acts as an mRNA for translation of the replicase polyproteins required for viral replication [14].

Coronaviruses have been reported to predominantly reside in an animal reservoir, such as bats, mice, rats, chickens, dogs, cats, horses, and camels [1,20]. Recently, the virus has developed the ability to initiate an epidemic by adapting to humans via zoonotic transmission, similar to the previous Zika virus outbreak in 2015 [21,22,23]. Bats have been reported to be the primary carrier and reservoir for a vast range of viruses, including the coronavirus, thus making the animal–human species barrier cross highly probable due to the large number of bats that congregate within the community and their ability to travel long distances [24]. Human coronaviruses were first discovered in the 1960s [25]. To date, studies have reported seven different strains of human coronaviruses. The four common coronavirus strains, including 229E, NL63, OC43 and HKU1, are known to cause mild respiratory tract infections worldwide [26,27]. Coronaviruses previously known to infect animals may evolve and adapt to infect humans, thus resulting in the emergence of a novel virus and the possibility of a pandemic outbreak [28]. The SARS-CoV, MERS-CoV and the more recent SARS-CoV-2 are examples of viruses crossing the animal-to-human species barrier and are known to cause more severe symptoms in patients [29]. As shown in Table 1, although the number of SARS-CoV-2 positive cases has well surpassed the number of MERS-CoV and SARS-CoV cases, the fatality rate indicates that the MERS-CoV and SARS-CoV were more likely to cause death in an infected individual.

In general, RNA viruses, such as the coronavirus, influenza virus and HIV, are known to have extremely high mutation rates due to their replication mechanism and the lack of viral RNA polymerase proofreading activity [35,36]. Mutations are the building blocks of evolution, allowing natural selection for traits beneficial to the virus, such as enhanced virulence, adaptability and evolvability [37]. According to a phylogenetic study by Lu et al. [27], SARS-CoV-2 was reported to have jumped the species barrier from bats sold at the Huanan South China Seafood Market in Wuhan, Hubei Province, China. Furthermore, the study reported higher SARS-CoV-2 genomic sequence similarity to the SARS-like bat coronaviruses RaTG13 (96.2% identity) collected from bats in Yunnan as compared to the SARS-CoV (79%) or MERS-CoV (51.8%), thus suggesting bats as the primary source of SARS-CoV-2 (see Table 2). Due to the large variety of animals being sold in the Huanan Seafood Market, researchers speculate an intermediary animal, such as snakes, pangolins, birds and other mammals, might have also played a role in facilitating the emergence of the current COVID-19 outbreak [27,32,38].

A recent study by Tang et al. [41] suggested that the SARS-CoV-2 is continuously evolving. The study compared the genome of SARS-CoV-2 isolated from 100 patients, including 73 patients from Wuhan and 27 patients from outside Wuhan. A total of 149 mutation sites were identified across the 100 genomes studied, with two SNPs found to exhibit strong linkage suggesting two different SARS-CoV-2 subtypes. The first SNP was identified at 8782 (orf1ab: T8517C, synonymous) whereas the second was identified at 28,144 (ORF8: C251T, non-synonymous—S84L). The genomes were then categorized based on the second SNP, which either had a serine (S) or leucine (L) at amino acid 84 of ORF8. Subsequently, genomic alignment to related viruses revealed the S type as the ancestral type, although the L type was found to be more prevalent, particularly in Wuhan.

The coronavirus spike protein, a viral surface protein known to play a significant role in viral attachment and host cell entry, is an interesting target for evolutionary studies due to its role in conferring host selectivity and susceptibility and viral infectivity [42,43,44]. In addition, being the major target of the host immune system, the spike protein has been reported to constantly undergo rapid molecular evolution and selective pressure [45]. A study by Zhang et al. [46] reported the emergence of the D614G mutation in the SARS-CoV-2 spike protein, which resulted in reduced S1 shedding and increased infectivity. It is suggested that further studies on SARS-CoV-2 spike evolution be conducted through epidemiological surveillance in order to understand the association between SARS-CoV-2 mutations and virulence.

## 3. COVID-19 Transmission

### 3.1. Animal-to-Human Transmission

**Figure 1:**
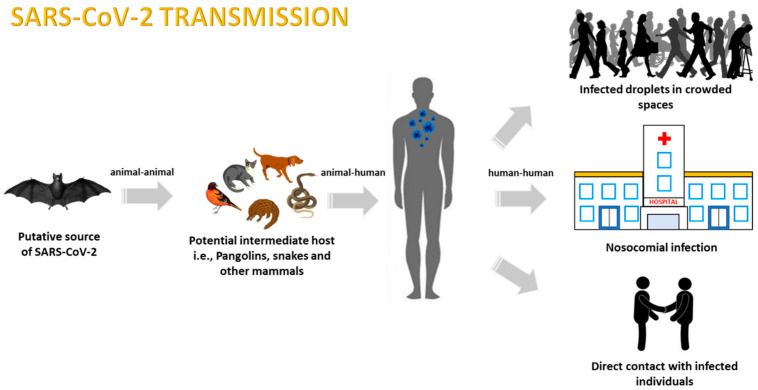
Representation of the zoonotic COVID-19 modes of transmission.

As recently determined, COVID-19 transmission has been identified to originate from bats but may have been transmitted to humans through other intermediate animals potentially sourced from the local seafood market in Wuhan city, Hubei province, China [47]. A study performed by Xiao et al. [48] mentioned that for SARS-CoV-2 to transmit to humans, an intermediate host must always be present, as bat-derived CoVs rarely infect humans. The study also reported that Chinese and Malayan wild pangolins were tested for SARS-CoV-2-like coronaviruses, with a majority testing positive. It is important to highlight that after thorough analysis, a single receptor-binding domain (RBD) in the spike protein of the Pangolin-CoV was found to have a minor difference in only one amino acid from that of SARS-CoV-2. These data further indicated that the SARS-CoV-2 may have potentially originated from the viral recombination between Pangolin-CoV and Bat-nCoV before transmitting to humans [48] (Figure 1).

### 3.2. Human-to-Human Transmission

#### 3.2.1. Transmission via Aerosols

Although multiple reports have mentioned that a carrier must be present for SARS-CoV-2 to transmit, there are additional forms of viral transmission that have been observed throughout this pandemic [1,49,50]. A study recently performed by Chan et al. [47] reported the case reports of a family of six patients who have been tested positive for the SARS-CoV-2, including their contact-tracing history and epidemiological, clinical, radiological and microbiological findings. Of the six family members, one member, who had not travelled to Wuhan city and yet had tested positive for SARS-CoV-2 after close contact with the family members, was among the first indications of positive person-to-person transmission of COVID-19 [47]. Primarily, it is now recognized that the main form of human-to-human transmission occurs through respiratory droplets expelled by an infected individual; hence, coughing and sneezing renders SARS-CoV-2 airborne, putting non-infected individuals at risk of contracting the disease [51,52]. Additionally, data have indicated that SARS-CoV-2 transmission can also occur as a result of contact with contaminated inanimate objects, also known as fomite transmission [53].

#### 3.2.2. Nosocomial-Related Infections

Hospitals are known to be one of the sources of secondary SARS-CoV-2 transmission, as they host a large number of infected individuals [54]. In the case of COVID-19, viral contamination in hospital rooms where COVID-19 patients are being cared for has been reported as another mode of transmission. In a recent study by Santarpia et al. [55], surface samples from the wards of SARS-CoV-2 positive patients were collected for evidence of viral RNA, and it was found that common items, such as toilet facilities, as well as air samples, tested positive for SARS-CoV-2 [53,55]. Among the variety of samples taken in this study, the highest airborne viral concentrations obtained originated from patients who were receiving oxygen through a nasal canula, with the results being 19.17 and 48.22 copies of virus per litre of air [55].

As SARS-CoV-2 spreads through droplets and fomites, it is important that healthcare sectors carefully consider methods to incorporate into practice so as to control the potential transmission within nosocomial environments. Aside from decontamination of common areas, equipment, and self-protection, precautionary controls can be taken to avoid potential spread during medical procedures. As SARS-CoV-2 is found in respiratory droplets and abundantly present in nasopharyngeal and salivary secretions, considerations when performing oral procedures such as endoscopies and dental care are required [52]. Ather et al. [52] discussed a step-by-step implication process that could be used for patient management and screening before dental procedures [56]. More recently, it has been highlighted that SARS-CoV-2 has been detected in the fecal samples of infected patients, indicating the ability of SARS-CoV-2 to proliferate within the digestive tract and the potential for fecal–oral route of transmission [57,58]. A study by Xiao et al. (2020) has found that when Vero E6 cells are inoculated with fecal samples from infected individuals, they present a cytopathic effect two days after a second-round passage [59]. Additionally, when the culture was visualized using electron microscopy, whole spherical viral particles with distinct surface spike projections were visible, indicating further proof of present virus progenies after a second-round passage of the cells [59]. An analysis of data of Hong Kong COVID-19 patients found that 17.6% of patients with the disease had gastrointestinal symptoms. In addition, 48.1% of patient stool samples tested positive for virus RNA even when the respiratory samples tested negative. This cohort study advises that health care workers exercise caution during patient fecal sample collections and when performing other medical procedures [60]. Therefore, additional care must be taken when performing medical procedures such as colonoscopies, as contact with patient fecal samples may occur [56].

Hospital intensive care units (ICU) have come across challenges in the rise of the COVID-19 pandemic. Alongside the urgency to meet the healthcare demand, another challenge has been to minimize the transmission of the virus from COVID-19 ICU patients to other patients and hospital practitioners. To overcome this, measures have been recommended and applied to ICU regulations, including managing ICU capacity, staffing workload and infrastructure for infection prevention [61]. Clinical management of critically ill COVID-19 patients requires adequate care to be given in disinfecting non-disposable items that come into contact with more than one patient, such as ventilators and ICU beds, so as to reduce the chances of fomite transmission [61].

#### 3.2.3. Maternal Transmission

Intrauterine vertical transmission potential from pregnant mothers to their children has been reported to be negative in nine SARS-CoV-2 positive pregnant mothers. All nine newborn children were tested for the SARS-CoV-2 and were reported to be negative for the virus. In addition, breast milk samples, amniotic fluid and cord blood collected from the patients also tested negative for the virus [62].

## 4. COVID-19 Modes of Detection

Based on our literature search, it has come to light that manifestations of COVID-19 differ greatly in patients and are inconsistent across different patients. It is important to note that early detection is a hugely important step that is required to be taken when diagnosing a patient during this pandemic [63]. It is not uncommon that patients are dismissed early on before COVID-19 is diagnosed. The pattern of this disease is that it lies undetected (whether dormant or in little amounts) for certain periods of time. In many observed cases, SARS-CoV-2 was not detected in nasopharyngeal/sputum samples. Although it is a challenge for hospitals to keep patients for long periods of times, it is crucial that each patient is given the benefit of multiple tests and monitored for even the slightest symptoms. As COVID-19 has presented its severity primarily in the form of respiratory distress, other symptoms such as abdominal distress may be dismissed as arising from other causes [64]. It is to be stressed that patients admitted to the hospital for other causes besides the common COVID-19 symptoms could still be carriers or positive for SARS-CoV-2 and thus clinical management is crucial for all hospital patients to minimize transmission [65].

With the recent widespread outbreak of COVID-19 across the globe, there has been an increasing need for the rapid, cheap and accurate detection of the virus in infected individuals. Suspected COVID-19 patients admitted to hospitals are usually required to provide biological samples, such as sputum/lower respiratory tract samples, nasopharyngeal swabs, blood and fecal samples, for diagnostic purposes [47]. In the earlier laboratory testing guidelines, the WHO advised medical teams to preliminarily screen suspected patients for common viruses and bacteria known to cause respiratory illnesses. The Xpert Express Flu/RSV assay is commonly used to test against 18 respiratory illnesses, including influenza A and B [47]. Negative results for rule-out screens require patient samples to be sent to a regional, national or international reference laboratory for new pathogen discovery [66]. The WHO continuously updates the gold standard for COVID-19 testing as more robust techniques are being developed for COVID-19 diagnosis [67]. In general, the first line of screening involves the detection of viral genomic material through reverse transcription polymerase chain reaction (RT-PCR), followed by complementary radiological and serological testing [68,69]. Overall, the field of SARS-CoV-2 detection is rapidly advancing towards improving accessibility and scalability while reducing bottleneck challenges such as assay costs, supply chain shortage and complexity [70].

### 4.1. Reverse Transcription Polymerase Chain Reaction (RT-PCR)-Based Testing

Whole genome sequencing analysis of the novel coronavirus has been performed recently and has allowed the analysis and selection of genes that are specific to the virus. This allows accurate and precise testing to be performed for the confirmatory diagnosis of infected patients using conventional real-time or RT-PCR. With this information, Chan et al. [47] developed and optimized primers for RT-PCR for the detection of the spike gene (S) that is specific only to the novel coronavirus. The forward and reverse primers are as follows: (5′-CCTACTAAATTAAATGATCTCTGCTTTACT-3′) and (5′-CAAGCTATAACGCAGCCTGTA-3′), respectively. In addition, a study by Corman et al. [69] reported primers specific to other genes of the novel coronavirus, such as the RdRp gene, the E gene and the N gene. Any of the following patient samples—saliva, respiratory, stool, urine or serum or plasma—are adequate to use for viral RNA isolation with this method. The detailed conditions for this assay can be found in the published article by Chan et al. [47]. More recently, a protocol for simple, accelerated and sensitive detection of SARS-CoV-2 using saliva samples, known as the SalivaDirect assay, has received emergency use authorization from the Food and Drug Administration [70].

### 4.2. Radiological Testing

Radiological imaging using computerized tomography scanning (CT scan) of the lungs of patients has revealed patterned characteristics that are now being used as a complementary diagnostic tool in hospitals. The chest images of novel coronavirus-infected patients show ground-glass opacities in certain lung segments and are now considered common CT findings of novel coronavirus pneumonia patients [47,71]. Indications of bilateral ground-glass opacities and the observation of lung consolidation should be a prompt for immediate tests against COVID-19 [72]. Chest CT images have been reported to provide 98% sensitivity in screening for SARS-CoV-2 infections. In a recent study by Fang et al. [73], the sensitivity of 51 patient chest CTs were compared to the results of RT-PCR, and it was found that chest CTs were able to detect evidence of abnormalities, indicating viral pneumonia in patients even when their RT-PCR results were initially negative. The authors discussed that even though RT-PCR is a highly sensitive method for SARS-CoV-2 detection, certain shortcomings may lead to negative results, such as improper clinical sampling or low patient viral load. This highlights the importance of using multiple detection methods in order to ensure accurate patient diagnosis.

In light of the accuracy and abundance of chest CT images available, artificial intelligence (AI) has been widely discussed and is in the early stages of application for the enhancement of SARS-CoV-2 detection. Although not all AI sources are available to most researchers, some have been described as open source and available to the public. COVID-Net is a deep conventional neural network that has been designed to recognize the chest X-ray (CXR) images of SARS-CoV-2-positive patients [74]. A study by Li et al. [75] used COVNet, a 3D deep learning model that extracts visual features from chest CT images to aid in the detection of SARS-CoV-2 infection. This model has been shown to accurately distinguish between COVID-19, community-acquired pneumonia and other lung disease presentations, further advancing the detection methods associated with the COVID-19 [75].

### 4.3. Serological and Immunological Assays

Serological testing involves the use of serum sample from a patient to screen for any immunologic responses that are commonly seen to change under the influence of a specific infection. Currently, serum samples are recommended to be tested in pairs, with the first sample collected during the first week of illness and the second collected three to four weeks after, which is necessary for confirmation of the disease [67]. Patients in severe and moribund conditions have shown serum profiles with drastically increased plasma concentrations of interleukins, including IL-6, IL-2, IL-7 and IL-10. In addition, it has been reported that COVID-19 critical patient serum profiles associate with a “cytokine storm” of immune factor upsurge [76]. In patients who do survive this extreme immune response, long-term effects, including lung damage or fibroses, have been shown to follow. Patients who are diagnosed with underlying chronic conditions have been additionally vulnerable to novel coronavirus infection as compared to patients with a healthy immune system [77]. Other blood profiles associated with COVID-19-ill patients include leucopenia, high C-reactive protein (above 10 mg/L), high erythrocyte sedimentation rate and elevated D-dimer [71].

#### 4.3.1. COVID-19 Testing Kits

With the urgent requirement for rapid and robust COVID-19 testing, multiple companies have developed COVID-19 test kits that are either being purchased directly by individuals in the community for home-based testing or are being used in medical laboratories to complement RT-PCR and radiological test results (Table 3). Although not as accurate as RT-PCR tests, COVID-19 rapid test kits have a turn-around time of under one hour as compared to RT-PCR tests, which may take up to two days [78]. Kits also allow for point-of-care testing as well as the ability to immediately test an individual who is suspected of carrying the SARS-CoV-2. Rapid detection kits also increase the ability to test a larger portion of the population, thus further aiding in outbreak control and accurate determination of the disease fatality rate. Besides detecting individuals with ongoing SARS-CoV-2 infections, COVID-19 test kits also aid in detecting asymptomatic individuals who have developed immunity against SARS-CoV-2, as detected through IgG and IgM molecules [78,79].

#### 4.3.2. CRISPR-Based Detection Techniques

Two pioneer companies, namely Mammoth Bioscience and Sherlock Biosciences, have been independently working on the development of CRISPR-based COVID-19 detection kits. Sherlock Biosciences has developed the technology through the SARS-CoV-2-specfic guide RNA activation of Cas13, which subsequently cuts a reporter RNA sequence [96]. On the other hand, Mammoth Biosciences utilized Cas12a, which is designed to be activated by SARS-CoV-2 E and N gene sequences [96]. More recently, the Council of Scientific and Industrial Research in India also announced the successful development of a CRISPR-based detection system known as the FNCAS9 Editor-Linked Uniform Detection Assay (FELUDA), which reduces the testing duration down to 30 min [97,98]. The CRISPR-based methods do not require complex instrumentation for testing and, in fact, the technology has been transferred onto paper strips, thus allowing for ease of distribution and diagnosis. The CRISPR-based kits have been reported to be robust (results obtained under one hour), economical and have high sensitivity and specificity [96].

## 5. COVID-19 Control Measures

### 5.1. Antiviral Drug Therapeutics

#### 5.1.1. Repositioning Antiviral Drugs as Therapy for SARS-CoV-2

Prior experience in handling the SARS-CoV and MERS-CoV outbreaks allowed government officials and healthcare providers to be better prepared to take on and contain the current COVID-19 outbreak [99,100,101]. Treatment provided by healthcare practitioners is customized according to the symptoms and severity experienced by the patient. Currently, there are no existing licensed anti-viral therapies that are specific for COVID-19, and therefore there is an urgent requirement for the development of such treatments [102]. Aside from the usage of broad-spectrum antibiotics to tackle pneumonia resulting from secondary infection, there have been suggestions by researchers to treat patients using a combination of, or analogs of, already existing antiviral drugs. Specific targeted drugs for the treatment of COVID-19 will require long years of development for the evaluation of drug delivery safety, pharmacokinetics and side effects before it is ensured that they are safe for human consumption [102].

A few clinical trials are in the midst of screening existing antiviral drugs to identify the best-suited selection that could be specific to target COVID-19 [103,104]. Due to the current urgent need to treat COVID-19-positive patients, the use of already existing medications and antivirals is a good and safe option to potentially reduce disease severity. Depending on the conditions of the patient, some non-antiviral drugs, such as metformin, glitazones, fibrates, sartans and nutrient supplements, could aid in the reduction of immunopathology caused by infection and improve the patients’ condition by enhancing their immune systems and preventing or restricting the effects of acute respiratory distress syndrome (ARDS) [76]. It has been suggested that neuraminidase inhibitors commonly used for influenza, the protease inhibitors lopinavir and ritonavir used for human immunodeficiency virus (HIV), nucleoside analogues, remdesivir, RNA synthesis inhibitors like TDF/3DC and anti-inflammatory drugs be used as potential antiviral treatments for COVID-19 [103,105]. For example, a case study in Bangkok, Thailand reported the dramatic health improvement of a COVID-19 infected patient within 48 h after being administered with a combination of the antiviral drugs oseltamivir (influenza), lopinavir and ritonavir (HIV) [105]. In addition, according to a study by Huang et al. [106], COVID-19-positive patients diagnosed with pneumonia were prescribed with oral and intravenous antibiotics along with 150 mg of oseltamivir. Patients diagnosed with more severe pneumonia were administered between 40 and 120 mg of methylprednisolone corticosteroid. Additionally, nasal cannula and invasive mechanical ventilation methods were given for oxygen support to patients experiencing hypoxemia [106].

#### 5.1.2. Clinical Trials for Drugs against COVID-19

The process of repositioning already developed drugs for use in COVID-19 treatment has now been given serious consideration and multiple drugs are undergoing clinical trials for approval of administration against the disease. Remdesivir, a broad-spectrum anti-viral drug initially developed for treating Ebola virus infections, has been given much attention and was among the first drugs to be accepted for clinical trials against COVID-19 [107]. Evidence has shown that Remdesivir successfully reduced viral load in COVID-19 patients and reduced recovery time [107]. Baricitinib, an anti-inflammatory drug initially developed for rheumatoid arthritis, has also been identified as a potential therapeutic agent using artificial intelligence-derived knowledge graphs [108]. More recently, it was announced that clinical trials with over 1000 enrolled participants have begun for studying the effect of remdesivir administration in combination with baricitinib [109]. Baricitinib targets and inhibits members of the numb-associated kinase (NAK) family, such as AAK1 (a regulator of clathrin-mediated endocutosis), which may in turn inhibit viral infection in host cells [108]. Another promising drug that is under evaluation for COVID-19 is favipiravir (FPV), with preliminary clinical results providing proof of viral clearance and improvement in patient chest imaging [110]. Currently, a few countries such as Japan, China and the United States are conducting clinical trials for FPV [110,111].

#### 5.1.3. Repositioning Hydroxychloroquine as a Drug Therapy for SARS-CoV-2

Recent developments in the quest for a cure for COVID-19 include the consideration of using the already available antimalarial drug chloroquine or its alternative less toxic forms, hydroxychloroquine or chloroquine phosphate, as treatment. A study by Wang et al. [112] evaluated the in vitro effects of chloroquine against a clinical isolate of SARS-CoV-2 and has determined that it effectively controls viral load. A recent review adequately summarized the current available literature on the potential usage of chloroquine as a treatment for COVID-19 [113]. Additionally, a small study by Gautret et al. [114] has indicated the usage of hydroxychloroquine and azithromycin in combination to efficiently reduce viral loads in COVID-19 patients. The drug combination was tested on 20 COVID-19-positive patients, with the results suggesting significant reduction/disappearance of viral load in all patients. As a recent follow-up, an observation study by Geleris et al. [115], reported that the efficiency of hydroxychloroquine may not be as promising as initially expected. In their study, they observed 1446 patients, all COVID-19-positive, of which almost 60% received 600 mg of hydroxychloroquine twice daily. Their analysis suggested that there is no significant association between hydroxychloroquine and intubation or death [115]. Concluding remarks of this study stated that hydroxychloroquine requires additional controlled trials with COVID-19 patients to further identify whether it is indeed effective in improving patient conditions. The value of hydroxychloroquine in treating COVID-19 remains unclear. Additional studies are required to highlight and understand the antiviral mode of action of hydroxychloroquine.

In summary, the current recommended drug treatment strategy for the treatment of COVID-19 is through repurposing already available drugs/antivirals. The drugs that are under consideration include remdesivir, chloroquine and hydroxychloroquine, ritonavir/lopinavir [116] and FPV, which are all undergoing either observations, studies, and/or clinical trials.

#### 5.1.4. Host–Virus Interaction Studies to Identify Antiviral Molecules against SARS-CoV-2

In the recent years, targeting of host factors crucial for viral replication has also turned out to be an attractive, novel anti-viral approach [117,118]. High-throughput techniques, such as affinity-purification mass spectrometry, have allowed for the rapid identification of virus–host interaction partners [119]. In a recent publication by Gordon et al. [120], 332 high-confidence SARS-CoV-2-human protein–protein interactions were identified. Sixty-six of the identified host proteins were reported to have corresponding FDA-approved drugs, drugs in clinical trials and/or preclinical compounds available for efficacy testing against the SARS-CoV-2 infection. Using similar techniques, multiple studies have also been conducted in the past to identify SARS-CoV interaction partners. As shown in Table 4, the interaction between specific hosts and the SARS-CoV protein has been extensively studied to further provide insights into the molecular mechanisms of the viral infection as well as for developing broadly acting antivirals against the coronavirus. These interactions could serve as potential leads for studying the SARS-CoV-2–host interactions as well as for designing an effective antiviral molecule against SARS-CoV-2 infection.

### 5.2. Immunotherapy

High-dose intravenous immunoglobulin (IVIg) has been recognized as a therapeutic option for patients with severe COVID-19 [135]. A brief study by Cao et al. [135] documented improvement in the conditions of three COVID-19 patients after the administration of high-dose IVIg at 0.3 to 0.5 g per kg weight. Although the limiting factor of this study is that all three patients were administered different antiviral drug therapies, every patient in the study exhibited clinical improvement shortly after IVIg administration [135]. Controlled randomized studies using IVIg treatment are currently underway to further determine its effectiveness in treating COVID-19 [136]. It is important to note that IVIg therapy is not considered as a stand-alone treatment and is generally administered alongside other therapies for better patient recovery. Patients with critical conditions and those who are ventilator-dependent are among those who are under IVIg trials, in hopes of reducing the number of ventilator-dependent days [137]. Preliminarily, it is seen that treatment of critically ill COVID-19 patients with acute respiratory distress syndrome (ARDS) showed improvement when administered convalescent plasma alongside other antiviral treatments [138]. In a preliminary study, four patients receiving mechanical ventilation support showed normalized body temperatures within three days and significant viral load decreases 12 days post-transfusion, further highlighting the potential effectiveness of this treatment [138].

The serology profile of patients with severe COVID-19 commonly include elevations in interleukin-6 (IL-6) and cytokine storms [139]. According to a study by Huang et al. [106], chemokine and cytokine analyses from COVID-19 patients revealed increased amounts of proinflammatory cytokines, such as IL-6, IL1β, IFNγ, IP10, and MCP1, in the serum. In addition, patients admitted to the ICU were found to have higher concentrations of GCSF, IP10, MCP1, MIP1A, and TNFα as compared to those not requiring ICU admission, thus suggesting an association between cytokine storm and disease severity [106]. Tocilizumab, a recombinant monoclonal antibody, has a high affinity with IL-6, which in turn prevents its binding to its original receptor, reducing the inflammatory response. Tocilizumab has been tested for its potential in curbing the COVID-19 symptoms and, as hypothesized, has successfully shown to improve patient conditions [139]. In this study it was found that 75% of patients required less ventilation dependency on average. Another study observed that COVID-19 patients with severe pneumonia who had been administered tocilizumab showed a reduction of the risk of invasive mechanical ventilation and death as compared to untreated groups [140].

### 5.3. Development of Vaccines

#### 5.3.1. Clinical Trials for Vaccines against COVID-19

The development of vaccines against COVID-19 during the current pandemic outbreak is urgently required and is essential to prevent infections, control the disease’s spread and limit recurrence [141]. Currently, an approved specific vaccine against SARS-CoV-2 is still unavailable due to the novelty of the virus and the time required for vaccine development and approval. Currently, there are approximately 218 candidate vaccines formulated against COVID-19, with 26 candidates in phase 1–3 trials [142]. Table 5 lists the major vaccine formulations against SARS-CoV-2 and their characteristics. A wide array of molecular platforms has been considered in the formulation of COVID-19 vaccines, including RNA- and DNA-based formulations, virus-like particle vaccines, purified inactivated formulations, protein-based formulations, and viral vector-based formulations [143].

In most but not all vaccines, particular attention is granted towards the SARS-CoV-2 spike protein (S) for vaccine development. In previous SARS and MERS vaccine research, it was reported that the S protein subunit of the virus was responsible for producing higher neutralizing antibody titers and better overall protection; therefore, more patents were granted to protein vaccines as compared to other vaccine types [141]. Although SARS-CoV-2 may differ from SARS and MERS, it is quite likely that the same strategy could be beneficial for SARS-CoV-2 vaccine development [141].

#### 5.3.2. Peptide Vaccine

Nandy and Basak [178] have remarked that based on historical events, traditional methods of developing antiviral vaccinations fail to tackle urgent requirements for surfacing viral infections such as COVID-19. It is recommended that alternative strategies be considered, such as the development of peptide vaccines that target specific epitope regions or multiple surface proteins of the virus. Computational system biology approaches are used to predict peptide sequences of immune cells against specific viral epitopes, which upon introduction to a host would induce the immune system to respond to the invading pathogen [179]. The epitopes are then tested for population human leukocyte antigen (HLA) and autoimmune response risks initially. Following that, any highly-ranked epitopes would be selected for further analyses of efficiency, side effects, range and other variables to ensure the epitopes selected are suitable for further consideration [180].

Recently, a study used a combination of immune T-cell and B-cell epitope predictions with molecular docking simulations to design a potential epitope-based peptide vaccine that would trigger a host immune response against the Chikungunya virus [181]. Similar work has been performed by Chakraborty et al. [182], where a multi-epitope loaded peptide vaccination was designed against the Japanese encephalitis virus (JEV) by reverse vaccinology and in silico study of B and T-cells against five JEV proteins (viz. E, prM, NS1, NS3 and NS5). Ahmed et al. [183] explored the genetic similarity between COVID-19 and previous SARS-CoV coronaviruses by obtaining existing immunological studies on SARS-CoV to form predictions for peptide vaccine design against COVID-19. They have identified epitopes derived from spike (S) and nucleocapsid (N) proteins that are identical to those of the COVID-19 proteins and suggested the potential of targeting these epitopes for near future vaccine development against COVID-19 [183].

Many researchers and multinational corporations are racing to develop a vaccine against the novel coronavirus. The quest for vaccine development could be enhanced using information obtained from bioinformatics technology. Epitope predictions that allow the narrowing down of vaccine targets have been made for COVID-19 using computational analysis of the physicochemical properties of the virus from the now available online sequence. According to a study by Joob and Wiwanitkit [184], the peptide 929EDEE932 contains an area with the highest epitope property for vaccine development. Studies such as these are constantly being conducted, proving the potential of this approach for the development of novel vaccines to target fast-evolving viruses.

#### 5.3.3. Challenges in Vaccine Development

The demand for a vaccine to overcome this pandemic is undoubtedly urgent; however, it is critical that key research priorities are met during the development and testing process. In vaccine development for the SARS coronavirus of 2003, one of the major challenges was the resulting undesired immunopotentiation (eosinophilic infiltration) post whole virus vaccination and spike protein vaccination [185]. Eosinophils are granulocytes that have the ability to mediate immunopathology in certain diseases such as bronchial asthma [186]. Pulmonary eosinophilia could be induced by certain vaccinations and lead to vaccine-induced hypersensitivity, which may be potentially life-threatening. In the past, SARS-CoV-1 vaccines were shown to induce pulmonary eosinophilia in animal studies involving ferrets, monkeys and mice [187]. This may be a recurring adverse effect in vaccinations of similar formulations under current testing and should be given consideration throughout the trials. Another major challenge is for patients who are pregnant and those who have underlying health conditions or immunocompromised systems. These patients require prevention most urgently, as they are more vulnerable to the disease, and yet it has been highlighted that they may be required to wait a longer time before being allowed to undergo clinical trials, as they are currently under the exclusion criteria to volunteer for these trials. Adverse effects of vaccinations may further complicate the conditions of these patients; therefore, it remains a concern throughout the clinical trials for all vaccines currently under trial. In general, most clinical trials estimate study completion dates towards the end of 2020. Although the rapid development of a vaccine is desirable by the public, it is important to highlight that careful measures must not be overlooked. Despite the variety of technologies now available and being used in the research and development of vaccines, the development of standardized assays for the evaluation of immune responses post vaccine trials are essential to assess the effectiveness and safety of all vaccines [188]. Multiple assays to test vaccine efficiency and safety exist; however, it has come to light that there is no existing standardized protocol implemented throughout all vaccine clinical trials thus far.

### 5.4. Nosocomial Infection-Related Control Measures

In addition to the safety guidelines for preventing SARS-CoV-2 nosocomial infections outlined in Section 3.2.2, it is also important to note that the symptoms of COVID-19 vary vastly across positive patients. Therefore, additional care must be taken within the hospital premises to avoid nosocomial infections through undetected COVID-19 patients. One example is the clinical case of a 71-year-old woman who was admitted preliminarily for gastrointestinal infection, tested positive for SARS-CoV-2 three days after admission and was only then moved to a negative pressure room [189]. This clinical case highlights the importance of monitoring COVID-19 through seemingly unrelated symptoms [180]. Implementation of the enhanced traffic control bundling (eTCB) system, a model proposed to enhance the process of limiting nosocomial transmission of COVID-19, may aid in breaking the community–hospital–community infection cycle [190].

### 5.5. Population Outbreak Predictions Using Computational Tools

Epidemiologic strategies are crucial in pandemic situations, as any form of cautionary safety measure may contribute to a reduction in the regional/global spread of the disease. Aside from the essential safety measures applied in most regions of the world, newer forms of population analysis are being integrated to aid epidemiologic studies and prediction studies in order to effectively control viral spread. By using already available data from previous viral outbreaks and current data surfacing from the COVID-19 outbreak, researchers have been able to better understand the disease pattern and hence provide more refined population control strategies. The potential for sustained transmission can be predicted using mathematical models that estimate how transmission varies over time [191]. Recent developments in artificial intelligence (AI) now play a significant role in predicting epidemic peaks in affected areas as well as determining the approximate outbreak control date [192]. Advanced models, such as the modified susceptible-exposed-infected-removed (SEIR) model, uses population migration data to derive information that allows predictions to be made on the potential outbreak severity that may arise. These data can also be supported further using machine-learning artificial intelligence, as discussed in the study by Yang et al. [192]. In the past years since AI’s arising, diverse studies have applied machine learning models in their research to test the accuracy and efficacy of the systems using past data. For example, in 2018, an AI approach was developed to forecast oyster norovirus outbreaks along the Gulf of Mexico, where 15 years’ worth of epidemiological data was input into the program. The artificial neural network (ANN-2 Day) model they developed was then able to reproduce 19 years of historical oyster norovirus outbreaks in the region with high sensitivity, specificity and accuracy [193]. In 2016, researchers in Singapore developed the least absolute shrinkage and selection operator (LASSO), a machine learning statistical model that forecasts potential infectious outbreaks in the region, such as dengue virus [194]. Although these algorithms and models are promising, improvements and refinements to the programs are continuously being made throughout the research to enhance the quality and accuracy of the obtained data. Nevertheless, it is possible to achieve a paradigm shift in population disease outbreak predictions using these tools, as they can act as supporting evidence when making regional outbreak control decisions.

## 6. Preventative Strategies

### 6.1. Daily Safety Guidelines

The CDC has published safety guidelines that could help in the prevention of infection in the public [195]. Most notably in the guide is avoiding close contact with infected individuals, staying at home if one is showing disease symptoms, frequently disinfecting the household and regularly used items and frequently washing hands. Although initially wearing of masks was only recommended for individuals with COVID-19 symptoms, healthcare workers and individuals in close settings with infected patients, it is now suggested that wearing a mask in public can effectively reduce the risk of COVID-19 transmission [195]. The WHO has also provided recommendations for infection prevention including basic information on thoroughly cooking meat and eggs, washing of hands and covering the mouth and nose when sneezing or coughing [196].

### 6.2. Preventative Measures Adoption According to Age Group

SARS-CoV-2 has been reported to infect individuals from all age groups; however, there is a higher mortality rate in the elderly (individuals over the age of 50) and those with prior health complications. According to a study by Daoust (2020) [197], elderly individuals are more vulnerable to COVID-19 and are disproportionally affected. The elderly are advised to minimize contact with individuals outside their household and to remain at home in order to reduce the risk of SARS-COV-2 infection [198]. In addition, it is suggested that government efforts towards overcoming the pandemic and reducing COVID-19-associated mortality rates should be efficiently strategized to protect the elderly [197]. Infants have also been reported to be able to contract SARS-CoV-2 [199]. A 3-month-old female patient was diagnosed positive for SARS-CoV-2 in early February 2020, after close contact with her grandmother who was confirmed positive for SARS-CoV-2. Another positive case of a newborn was a 17-day-old male, who contracted the disease via close contact with his SARS-CoV-2-positive parents [200]. However, research suggests that only about 2–5% of infants born to women positive for COVID-19 during delivery test positive for the virus after birth [201,202]. Therefore, the adoption of general guidelines for protection against COVID-19 outlined by the CDC should suffice in protecting the newborn [203]. The pediatric cases reported for COVID-19 child infection rates are much lower than adult cases, with only mild to no symptoms reported in children [107]. Children aged below 14 have been documented to be less susceptible to SARS-CoV-2 infection as compared to adults over the age of 20 [204]. Therefore, following the daily safety guidelines outlined by the CDC should aid in mitigating COVID-19 spread among children while extra precautions, such as those recommended for the elderly, should be adopted by adults to protect against the infection.

### 6.3. Travel and Airport Screenings

To help prevent the spread of the novel coronavirus, advisories outlining specific travel measures have been implemented globally. Many airports have set up screening stations in order to detect symptomatic travelers [205]. In some countries, like Japan, there has been the implementation of quarantine check points at airports and other entry points to the country [206]. Airline companies have published preventive measures for passengers travelling to and from China at this time [207,208]. A recent study by Quilty et al. [209] was published detailing the effectiveness of airport screening for detecting COVID-19-infected travelers. Most countries have implemented movement control and lockdown operations in order to minimize exposure of the general public to potential incoming carriers of COVID-19 [210]. These operations aimed to reduce the number of infections within the general population in order to control the spread of COVID-19 regionally and globally.

## 7. Conclusions

The recent COVID-19 outbreak in China has raised serious concerns about the threat it poses to the global public health. Despite its lower mortality rate as compared to previous coronavirus outbreaks, such as the 2002 SARS-CoV and the 2012 MERS-CoV outbreaks, the SARS-CoV-2 has shown patterns of higher transmissibility. To date, limited standardized techniques, including PCR-based detection of specific SARS-CoV-2 genes and complementary radiological and serological tests, are available to detect the disease. To increase testing capacity and allow for point-of-care COVID-19 infection detection, multiple robust kits have been developed and are currently in use in diagnostic laboratories and in home-based settings worldwide. Currently, there is an absence of specific vaccines and antiviral therapeutics against COVID-19. In the meantime, the demand for urgent COVID-19 control and prevention has led to testing the efficacy of existing approved vaccines and drugs known to be safe for human immunization/consumption, as vaccines and antiviral therapeutics designed specifically against the COVID-19 may take years before entering the market. In addition, it is suggested that future studies focus on utilizing host–virus interaction data obtained from high-throughput studies as well as the COVID-19 AI database to aid in repurposing currently available drugs.

## Figures and Tables

**Table 1:** Properties of SARS-CoV-2, MERS-CoV and SARS-CoV [8,18,19,30,31,32,33,34]. (* updated as of 8 October 2020).

	SARS-CoV-2 *	MERS-CoV	SARS-CoV
Outbreak Date	December 2019	April 2012	November 2002
Epicenter of Disease Outbreak	Wuhan, China	Jeddah, Saudi Arabia	Guangdong, China
Original/Intermediate Animal Reservoir	Bats, Pangolins and potentially other mammals	Bats/Camels	Bats/Masked palm civets
Number of Cases	36,401,583	2494	8096
Number of Deaths	1,060,567	858	744
Transmission Rate	3–4	<1	3
Fatality Rate	1.4%	37%	10%
Countries/Territories affected	214	27	26

**Table 2:** SARS-CoV-2 genomic sequence similarity to SARS-like coronaviruses, SARS-CoV and MERS-CoV.

Coronavirus Strain	Genomic Sequence Similarity to SARS-CoV-2	Reference
SARS-like coronavirus bat-RaTG13	96.2%	[21]
SARS-like coronavirus bat-SL-CoVZC45	88%	[21]
SARS-like coronavirus bat-SL-CoVZXC21	88%	[21]
SARS-CoV	79%	[39]
MERS-CoV	51.8%	[40]

**Table 3:** Recently developed COVID-19 test kits and their characteristics.

Name	Company	Date of Release	Sample Type	Target Antigen	Duration of Test	Country of Validation	References
COVID-19 IgG/IgM Point of Care Rapid test	Aytu Biosciences	March 2020	Whole blood/serum/plasma	IgG/IgM	2–10 min	China and United States	[80]
Wantai SARS-CoV-2 Ab Rapid Test	Beijing Wantai Biological Pharmacy Enterprise	March 2020	Whole blood/serum/plasma	IgG/IgM	15 min	China and Australia	[81]
Biolidics 2019-nCoV IgG/IgM Detection Ki	Biolidics Limited	March 2020	Whole blood/serum/plasma	IgG/IgM	<10 min	Singapore	[82]
MAGLUMI IgG de 2019-nCoV	Snibe Diagnostic	March 2020	Whole blood	IgG	600 tests per hour	Brazil, China and United States	[83,84]
m2000 SARS-CoV-2 assay	Abbott Core Laboratory	April 2020	Whole blood/serum/plasma	IgG	100–200 tests per hour	United States	[85]
Rapid SARS-CoV-2 antigen detection test	Sona Nanotech	April 2020	nasal or oropharyngeal swabs	S1 subunit	-	Canada and United States	[86]
COVID-19 Ag Respi-Strip	Coris Bioconcept	April 2020	Nasopharyngeal secretions	N protein	<15 min	Belgium	[79,87]
SGTi-flex COVID-19 IgM/IgG	Sugentech	April 2020	Whole blood/serum/plasma	IgG/IgM	10–15 min	South Korea and United States	[88]
INNOVITA 2019-nCoV Ab Test (Colloidal Gold)	Bioforge Solutions Pte Ltd.	April 2020	Whole blood/serum/plasma	IgG/IgM	<15 min	China, Singapore and United States	[89]
Shanghai LiangRun LionRun Diagnostic Kit for Antibody IgM-IgG of Novel Coronavirus COVID-19	Veredus Laboratories Pte Ltd.	April 2020	Whole blood/serum/plasma	IgG/IgM	<10 min	China and Singapore	[90]
DiagnoSure COVID-19 IgG/IgM Rapid Test Cassette	Grit Overseas Pte Ltd.	April 2020	Whole blood/serum/plasma	IgG/IgM	<10 min	China, Singapore and Netherlands	[91]
VITROS Immunodiagnostic Products Anti-SARSCoV-2 Total Reagent Pack	Ortho Clinical Diagnostics	May 2020	Serum/plasma	IgG/IgM/IgA	150 tests per hour	Singapore and United States	[92]
MP Diagnostics ASSURE^®^ SARS-CoV-2 IgG/IgM Rapid Test	MP Biomedicals Asia Pacific Pte Ltd.	May 2020	Whole blood/serum/plasma	IgG/IgM	<25 min	Singapore	[93,94]
Roche Elecsys Anti-SARS-CoV-2	Roche Diagnostics Asia Pacific Pte Ltd.	May 2020	Serum/plasma	N protein/IgG	18 min	Singapore	[95]

**Table 4:** SARS-CoV–host protein–protein interaction partners and their implications in the SARS-CoV life cycle.

Host Protein	Viral Protein	Effect of Host–Virus Interaction	Antiviral Property of Host Protein	Inhibitors of Host Proteins	References
Actin	Nucleocapsid (N)	Viral N protein recognizes host actin and induces apoptosis in COS-1 cells	Data not available	Cytochalasin D *	[121,122,123]
cyclin-cyclin-dependent kinase complex	Nucleocapsid (N)	N protein inhibits S-phase progression in mammalian cells	Pro-viral ^-^	Palbociclib ^ Abemaciclib * Ribociclib *	[124,125,126]
14-3-3	Nucleocapsid (N)	Nucleocytoplasmic shuttling of the N protein is mediated by 14-3-3	Pro-viral ^-^	R18 ^#^ Fusicoccin ^+^ Phenethyl isothiocyanate ^+^	[127,128,129]
CRM1	9b accessory protein (9b)	Nuclear shuttling of 9b is dependent on CRM1	Pro-viral ^-^	Selinexor * Isoniazid *	[130,131,132]
AP-1	3b accessory protein	3b protein induces the transcriptional activity of AP-1	Pro-viral ^-^	Arsenic trioxide *	[133,134]

* U.S. Food and Drug Administration approved; ^ Phase II clinical trial; ^+^ Experimental; ^#^ Data not available; ^-^ Host protein is essential for viral replication.

**Table 5:** Vaccine candidates undergoing clinical trial testing and their characteristics (updated as of 05/11/2020).

Vaccine Name	Company	Clinical Trial Phase/Identifier	Number of Participants Enrolled in Trial	Vaccine Dosage Received	Vaccine Type	Target/Mode of Action	Reported Adverse Effects	References
mRNA-1273	National Institute of Allergy and Infectious Diseases (NIAID) and Moderna	Phase III (NCT04470427) Phase I/II (NCT04283461)	30,000/600	100 μg (intramuscular)/50 μg or 250 μg (intramuscular)	Novel mRNA vaccine encapsulated in lipid nanoparticle for delivery	Encodes for a full-length stabilized form of the S protein	No obvious side effects	[144,145,146,147]
Ad5-nCoV	CanSino Biologics Inc and collaboration with National Research Council of Canada and Beijing institute of Biotechnology	Phase III (NCT04526990) Phase II (NCT04341389)	40,000/500	Low: 5 × 10^10^ vp Middle: 1 × 10^11^ vp High: 1.5 × 10^11^ vp (intramuscular)	Recombinant adenovirus type-5 vector to express spike protein	Adenovirus vector express SARS-CoV-2 spike protein	High dosage group experienced higher fever within 24 h of administration	[148,149,150,151,152]
AZD1222 (Formerly known as: ChAdOx1 nCoV-19)	Oxford University	Phase III (NCT04516746) Phase I/II (NCT04324606)	30,000/1090	Single dose of 5 × 10^10^ vp (intramuscular) Booster dose: 2.5 × 10^10^ vp	Chimpanzee adenovirus vector encoding spike protein of SARS-CoV-2	Elicits humoral and cell-mediated response against spike protein	Mild increase in temperature, headache, or sore arm.	[153,154,155,156,157]
Bacille Calmette-Guerin (BCG)	Multiple companies	Phase III (NCT04350931) (NCT04328441)	Variable	0.1 mL intradermal	Attenuated *Mycobacterium bovis*	Hypothesized that trained immunity would develop and may aid in immunity against COVID-19	To be assessed	[158,159,160]
Measels-Mumps-Rubella vaccine (MMR)	Kasr El Aini Hospital	Phase III (NCT04357028)	200	0.5 mL subcutaneous	Attenuated virus vaccine	Hypothesized that MMR may lower serological incidence caused by SARS-CoV-2 as neutralizing antibodies will be produced	To be assessed	[161]
BNT162 (a1, b1, b2, c2)	Biontech RNA Pharmaceuticals GmbH, Pfizer	Phase I/II (NCT04380701) Approaching phase III	7600	0.5 mL (intramuscular)	mRNA and lipid nanoparticle	mRNA encoding spike protein and receptor-binding domain gets delivered via lipid nanoparticles into host cells activating immunity against SARS-CoV-2	Fever, fatigue, vomiting, diarrhea, or worsened muscle pain	[162]
INO-4800	Inovio Pharmaceuticals Inc.	Phase I/II (NCT04447781) Phase I (NCT04336410)	160/40	Two doses of 1 mg each	Plasmid DNA that encodes spike protein	Host cells translate spike protein encoded by plasmid eliciting immune response against spike protein	To be assessed	[163,164,165]
COVID-19/aAPC	Shenzhen Geno-Immune Medical Institute	Phase I (NCT04299724)	100	Thee injections 5 × 10^6^ each (Subcutaneous)	Modified Lentivirus	Lentivirus vector presents modulatory and viral genes to artificial antigen presenting cells (aAPCs)	To be assessed	[166]
LV-SMENP-DC	Shenzhen Geno-Immune Medical Institute	Phase I/II (NCT04276896)	100	5 × 10^6^ (subcutaneous)	Modified Dendritic cells (DC) with Lentivirus	Modified DC with Lentivirus vector carries SMENP minigenes to express COVID-19 antigens to	To be assessed	[167]
V-SARS	Immunitor LLC	Phase I/II (NCT04380532)	20	Vaccine formulated as oral pill, one pill-per-day for 1 month	Heat-inactivated plasma from COVID-19 patients	Host immunity development against COVID-19. Exact mechanism to be determined.	To be assessed	[168,169]
AV-COVID-19	Aivita Biomedical, Inc	Phase I/II (NCT04386252)	180	Variable	Autologous DC loaded with SARS-CoV-2 antigens	Host immunity development against COVID-19. Exact mechanism to be determined	To be assessed	[170]
Inactivated SARS-CoV-2	Sinovac Research and Development Co., Ltd.	Phase I/II (NCT04352608)	744	Medium dose: 600 SU/0.5 mL High Dose: 1200 SU/0.5 mL	Inactivated SARS-CoV-2 virus	Inactivated whole virus yields immunization through producing IgG against viral spike–receptor binding domain	To be assessed	[171]
GX-19	Genexine, Inc.	Phase I/II (NCT04445389)	210	Not revealed. (Intramusculary)	DNA vaccine	DNA vaccine which expresses SARS-CoV-2 Spike protein antigen	To be assessed	[172]
SARS-CoV-2 rS or NVX-CoV2373	Novavax	Phase I (NCT04368988)	131	25 μg without Matrix-M, 5 μg with 50 μg Matrix-M	Nanoparticle vaccine with/without Matrix-M adjuvant	Efficient binding with viral targeted receptors. Adjuvant stimulates high levels of neutralizing antibodies	To be assessed	[173,174]
bacTRL-Spike	Symvivo Corporation	Phase I (NCT04334980)	84	Vaccine formulated as oral pill. Dosage variable	Bacterial vaccine	*Bifidobacterium longum* delivers synthetic plasmid DNA containing spike protein of SARS-CoV-2	To be assessed	[175]
SCB-2019	Clover Biopharmaceuticals AUS Pty Ltd.	Phase I (NCT04405908)	150	3–30 μg twice daily (intramuscular)	Recombinant subunit vaccine	Synthesized subunit that resembles viral spike protein induces immunity against spike protein of SARS-CoV-2	To be assessed	[176,177]
